# The Pak4 Protein Kinase in Breast Cancer

**DOI:** 10.5402/2012/694201

**Published:** 2012-12-27

**Authors:** Audrey Minden

**Affiliations:** Susan Lehman Cullman Laboratory for Cancer Research, Department of Chemical Biology, Ernest Mario School of Pharmacy, Rutgers, The State University of New Jersey, Piscataway, NJ 08854, USA

## Abstract

Paks4, along with Paks5, and 6 are members of the group B family of p21-activated kinases (Paks). The Paks play multiple different roles in controlling cell morphology, cell growth, proliferation, and signaling. Pak4 has essential roles in embryonic development (Qu et al., 2003), but in adults high levels of Pak4 are frequently associated with cancer. Pak4 has been implicated in several types of cancer (Wells and Jones, 2010; Eswaran et al., 2009; Liu et al., 2008; and Liu et al., 2010) and it is strongly linked to breast cancer (Liu et al., 2008; Liu et al. 2010; Yu et al., 2009; Rafn et al., 2012; and So et al., 2012). Breast tumors and breast cancer cell lines frequently have high levels of Pak4 (Liu et al., 2008), and overexpression of Pak4 in mammary epithelial cells leads to tumorigenesis in mice (Liu et al., 2010). This paper summarizes the current work on the role of Pak4 in breast cancer.

## 1. Introduction

**Figure 1:**
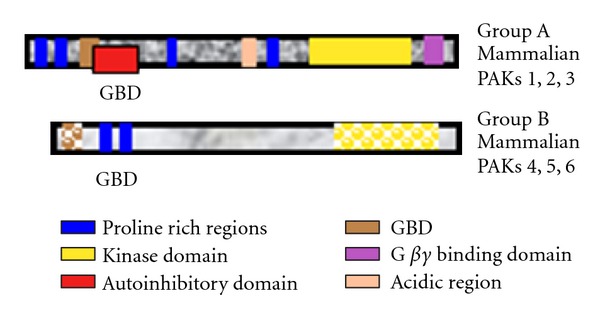
Schematic diagram of the structures of the group A and group B Pak family members.

The p21-activated kinase (PAK) family of serine/threonine kinases have important roles in cytoskeletal organization, cell signaling, and cell proliferation and survival [1, 2]. They were first identified as effector proteins for Cdc42 and Rac, members of the Rho GTPase family, but they can respond to many different types of signals. The Paks fall into two categories, group A and group B, based on their sequences and functions (see Figure 1).

The group A and group B Paks share in common an amino terminal GTPase binding domain (GBD) and a carboxyl terminal serine/threonine kinase domain, as illustrated in Figure 1. The GBD and kinase domains of the two groups, however, have only approximately 50% identity with each other, and the regulatory domains outside of the GBD and kinase domains are completely different in the group B Paks compared with the group A Paks. The different Paks also differ in their substrate specificity, although there is also some overlap [3, 4]. The different Pak family members differ in their expression patterns. Pak4 expression is high throughout the embryo during the development, but in many adult tissues Pak4 protein levels are low. Pak4 has an important role in embryonic development [5], but in adult tissues Pak4 overexpression is often associated with cancer. This paper will focus on Pak4, and recent studies aimed at investigating its role in breast cancer.

## 2. Pak4 and Breast Cancer

Numerous studies point to a role for the Pak kinases in oncogenic transformation [6–15]. Among the group B Paks, Pak4 is most closely linked to cancer, and it is overexpressed in many types of tumors and cancer cells [1, 6, 16–18]. In cells, Pak4 has been linked with many hallmarks of tumorigenesis, including anchorage independent growth [4, 19, 20], increased cell survival [9, 10], migration, and invasion [13, 21–24]. In mice, Pak4 overexpression leads to tumor formation in xenograft studies [7, 8].

In breast cancer, the link with Pak4 is quite striking. Pak4 is overexpressed in breast cancer cell lines [7, 8, 20], as well as in primary human breast tumor and rat mammary tumor samples [7], but it is barely detectable in normal tissue [7]. In the MCF10A cell progression series, which consists of mammary epithelial cells that range from nontransformed to highly tumorigenic, Pak4 levels are higher in the more tumorigenic cells [25]. The chromosomal region containing Pak4, 19q13.2, is frequently amplified at a high rate in aggressive breast cancers with basal-like features [26].

The Pak4 expression pattern—high in breast tumors but low in normal breast tissue—could make Pak4 a promising diagnostic tool for the disease. In addition to serving as a marker for cancer, however, Pak4 can also cause mammary tumors to form, and thus it has a strong potential as a drug target. The role for Pak4 in disrupting the normal structure of the mammary gland has been examined *in vitro* by studying the mouse mammary epithelial cell line iMMEC [8]. When grown in culture under conditions where they can grow into 3 dimensional structures, iMMECs form spherical acini with hollow lumens. These structures recapitulate the acinar subunits of normal mammary epithelium in many respects, particularly the hollow lumen surrounded by polarized epithelial cells. As such, these cells provide a 3D *in vitro* model for normal breast epithelium [27]. Furthermore, just as normal breast epithelia has nearly undetectable levels of Pak4, the normal iMMECs also have almost negligible amounts of Pak4 protein.

Strikingly, when iMMECs are stably transfected with wild-type Pak4, they take on quite dramatic changes [8], most of which are associated with oncogenic transformation. When Pak4 overexpressing iMMECs are grown in standard 2-dimensional culture conditions, the cells appear similar to the control cells, and no changes in cell proliferation are observed. However, when cells are grown under 3D culture conditions on a layer of basement membranes, important differences are observed. While acinar structures continue to form, they take on very different characteristics when Pak4 is overexpressed. The acinar structures increase in size, and, instead of a single layer of cells surrounding a lumen, there are multiple layers of epithelial cells. Further investigation shows that this is associated with an increased level of cell proliferation. Another important change is in the development of the hollow lumen. Normal iMMECs form a hollow lumen in the acinar structure, a process that is associated with apoptosis in the cells in the interior of the structure. When Pak4 is overexpressed, however, the lumen never gets completely hollow, and staining with active caspase-3 reveals that this change is associated with a drastic decrease in the level of apoptosis. This is consistent with Pak4's role in cell survival and inhibition of apoptosis, seen in several different types of cells [9–11]. Finally, the Pak4 overexpressing cells appear highly disorganized with respect to cell polarity. Normal mammary epithelial cells are highly polarized, with an apical membrane facing the lumen and a basolateral membrane facing the basement membrane that surrounds the acinar structures. In contrast, in the presence of Pak4, the epithelial cell membranes are disorganized, and the normal apical/basolateral structure is disrupted [8]. All of these changes seen in response to Pak4 are reminiscent of changes seen in the glandular epithelium during precancerous conditions and early tumorigenesis. Some of the changes, such as filling of the luminal space, are particularly reminiscent of atypical hyperplasia and DCIS [28]. These results suggest an important role for Pak4 in mammary tumorigenesis, but the ultimate test for tumorigenesis is tumor formation in mice. To test this, Pak4 overexpressing iMMECs have been implanted into the mammary fat pads of mice. The result is the formation of mammary tumors at a high frequency [8], indicating that Pak4 can be a driving force in oncogenic transformation of these cells.

These results indicate that Pak4 is sufficient to trigger oncogenic transformation in mammary cells. Other oncogenes, however, are also well known to be critical in breast cancer. In particular, the oncogene Her2Neu has an important role in the disease [29]. Her2Neu as well as oncogenic Ras also causes iMMECs to produce abnormal acinar structures and to form tumors in mice [27, 30]. It is important to note that in Her2Neu and Ras transformed iMMECs, Pak4 is also highly upregulated [8]. Pak4 has also been shown to play an important role in Her2Neu signaling [31]. Pak4 thus appears to be a key downstream target by which oncogenes promote mammary tumorigenesis.

One interesting result from the study described above is the finding that Pak4 overexpression disrupts cell polarity [8]. Alterations in cell polarity can be important in cancer, and the role for Pak4 in polarity could help explain how Pak4 could cause cancer. It is not yet clear how Pak4 can alter cell polarity when it is overexpressed. It is interesting that Pak4 along with Par6bs has recently been shown to be required for regulation of apical junction formation by Cdc42 in human bronchial epithelial cells [32]. Par6 is a cdc42 binding protein, known to play a role in cell polarity [33, 34]. Its binding to Cdc42 is important for establishing cell polarity, and one possibility is that Pak4 may interfere with the Cdc42:Par6 interaction when it is overexpressed, thereby disrupting cell polarity in cancer. The exact role for pak4 in cell polarity, and possible downstream mediators, is an area that needs to be explored further.

## 3. Other Pak Family Members in Breast Cancer

Other Pak family members have also been linked with breast cancer, especially Pak1. Pak1, however, appears to function differently from Pak4 both in cell culture and animal models of the disease. Transgenic mice have been generated that express a Pak1 mutant (Pak1T423E) in the mammary gland. Pak1T423E is a point mutant which has a constitutively high level of kinase activity. These transgenic mice develop mammary tumors, but at low penetrance and with a long latency period, suggesting that other genetic events are required in the transformation process [35]. An important difference between the Pak1 and Pak4 studies is that, in contrast to Pak1, even wild-type Pak4 leads to transformation of mammary epithelial cells and tumorigenesis in mice, and it leads to tumor formation at a high frequency [7, 8]. This is an important distinction, because so far point mutations in the Paks have not been frequently linked with cancer, but overexpression seems to be more significant. The distinctions between the different Paks are interesting, but at the same time it can be difficult to compare the Pak1 and Pak4 results because different types of conditions were used for the different studies. In the future it will be important to sort out the specific contributions of each of the Pak kinases in cancer.

Pak1 has been specifically linked to ErbB2 signaling in estrogen receptor (ER) negative tumors. Pak1 was shown to be activated in breast cancer cells that come from ER negative tumors that overexpress the ErbB2 oncogene [36]. Blocking Pak1 activity, in contrast, inhibits transformation of MCF10A cells by ErbB2 and blocks tumor formation in mice in response to ErbB2 positive breast cancer cell lines. The activated Pak1 mutant can bypass the requirement for ErbB2 activity in transformation [36]. These results suggest an important role for Pak1 in the ErbB2 pathway in breast cancer.

An interesting cell culture model for breast cancer is the MCF10A progression series. MCF10A, neoT, ATI, and DCIS cells are all derived from MCF10A cells. MCF10A represent normal human breast epithelium [37], similar in many respects to the mouse iMMECs. The other cells in the progression series are models for increasing levels of oncogenic transformation [38–40]. When grown in 3D culture, there is an increasing derangement of normal acinar structure in the more malignant cells. Pak4 levels increase in the more malignant versions of the cells [25]. Likewise, Pak1 expression and phosphorylation levels were also shown to increase in the more malignant versions of the cells [41], and the abnormal morphologies can be partially reversed by the expression of dominant negative Pak1. Overexpression of exogenous wild-type or activated Pak1, however, has no effects on cell proliferation, invasion, or acinar growth [41]. In contrast, as described above, overexpression of wild-type Pak4 has striking effects on mouse iMMECs, leading to alterations in acinar structure and tumorigenesis in mice [8]. 

## 4. Pak4 as a Drug Target

Pak4 has a great potential as a druggable target for the treatment of breast and other cancers. In fact, an inhibitor that blocks the kinase activity of Pak4 and other Pak family members (PF-3758309) has growth inhibitory activity towards a large number of tumor cell lines [42, 43], and a newer Pak4 inhibitor, LCH-7749944, has also been reported recently [44]. This potent Pak4 inhibitor affects several cell signaling pathways. Specifically, it downregulates a pathway mediated by Pak4/c-Src/EGFR and cyclin D1, and it inhibits EGFR activity. LCH-7749944 also affects cell morphology, by blocking filopodia formation and leading to cell elongation. This may be due to the fact that it blocks the cofilin pathway, which is tightly linked to cytoskeletal organization, as well as the ERK/MMP2 (matrix metalloproteinase) pathways. Importantly, this inhibitor suppresses proliferation of human gastric cancer cells and blocks their migration and invasion capacity. In order for Pak4 to be a fully effective drug target, however, better understanding of the mechanisms by which it causes cancer may be needed. One area of particular importance is the role for Pak4's catalytic activity. Most drugs that target protein kinases are designed to block kinase activity, but Pak4 and other Paks may have some kinase independent functions [9, 10, 45, 46]. For example, Pak4 has an important role in suppressing apoptosis, which could be directly related to its role in cancer, but under some conditions this occurs completely independently of Pak4's kinase activity [9–11]. Pak4 may thus serve not only as a protein kinase but it may also have other roles, which could include sequestering activities, scaffolding roles, and serving as a linker for protein complexes. The possibility that Pak4 could promote tumorigenesis by either a kinase independent mechanism or by a combination of kinase dependent and kinase independent mechanisms needs to be considered. In this case, drugs designed specifically to block Pak4 kinase activity alone may be incompletely effective in some types of cancer, and new strategies for blocking Pak4 should be investigated. Another important consideration is that more than one Pak family member may need to be targeted. The studies described above indicate that Pak1 is also linked to breast cancer, even though Pak1 and Pak4 probably function quite differently in the disease. The idea that both Pak1 and Pak4 could have roles in breast cancer has very important implications in the future drug development. It is therefore important to consider the need to develop ways to block multiple Pak kinases, in order to most effectively treat different types of breast cancer at different stages.

## 5. Conclusion

The Pak4 protein kinase is often overexpressed in breast tumors, but is poorly expressed in normal breast tissue. Pak4 is sufficient to transform noncancerous mammary epithelial cells and to form tumors in the mammary glands of mice. Other Pak kinases, particularly Pak1, have also been implicated in breast cancer, although the different Paks may function by different mechanisms. These findings suggest that the Pak kinases may be good targets for drugs designed to treat breast cancer. Better understanding of the molecular mechanism by which Pak4 and other Pak kinases promote cancer will be important for designing the best possible inhibitors.
